# Early maladaptive schemas impact on long-term outcome in patients treated with group behavioral therapy for obsessive-compulsive disorder

**DOI:** 10.1186/s12888-019-2285-2

**Published:** 2019-10-26

**Authors:** Tor Sunde, Benjamin Hummelen, Joseph A. Himle, Liv Tveit Walseth, Patrick A. Vogel, Gunvor Launes, Vegard Øksendal Haaland, Åshild Tellefsen Haaland

**Affiliations:** 1 DPS Solvang, Sørlandet Hospital, SSHF, Seviceboks 416, 4604 Kristiansand, Norway; 2 Clinic of Mental Health and Addiction, Oslo University Hospital, Oslo, Norway; 3 School of Social Work and School of Medicine-Psychiatry, University of Michigan, Ann Arbor, USA; 4 Department of Psychology, Norwegian University of Science and Technology, Trondheim, Norway; 5 Clinical Neuroscience Research Group, Department of Psychology, University of Oslo, Oslo, Norway

**Keywords:** Obsessive-compulsive disorder, ERP, Group therapy, Early maladaptive schema, Follow-up

## Abstract

**Background:**

Some studies have previously found that certain elevated early maladaptive schemas (EMSs) are negative predictors for outcome for patients with obsessive-compulsive disorder (OCD) treated with Cognitive-Behavioral Therapy (CBT) or Exposure and Response Prevention (ERP). The current study explores whether EMS were related to reductions in OCD symptom severity at long-term follow-up (*Mean* = 8 years) after group ERP for patients with OCD. The central hypothesis was that patients with no response to treatment or patients who relapsed during the follow-up period were more likely to have elevated pre-treatment EMSs compared to those who responded to initial treatment and maintained gains over time. We also investigated whether there were any differences in change over time of overall EMS between patients who were recovered versus patients who were not recovered at extended follow-up.

**Methods:**

Young Schema Questionnaire –Short Form (YSQ-SF), Yale-Brown Obsessive-Compulsive Scale (Y-BOCS), Beck Depression Inventory (BDI) were measured in 40 OCD patients in a general outpatient clinic before and after group ERP, after 12-months and at extended follow-up. To analyze the predictors, a multiple regression analyses was conducted. Changes in overall EMS was analyzed by mixed models procedures.

**Results:**

The major finding is that patients with high pre-treatment YSQ-SF total scores were less likely to respond to initial treatment or were more likely to relapse between post-treatment and the extended follow-up. The YSQ-SF total score at pre-treatment explained 10.5% of the variance of extended long-term follow-up outcome. The entire sample experienced a significant reduction in overall EMS over time with largest reduction from pre- to post-test. There were no statistically significant differences in total EMS change trajectories between the patients who were recovered at the extended follow-up compared to those who were not.

**Conclusion:**

The results from the present study suggest that patients with higher pre-treatment EMSs score are less likely to recover in the long-term after receiving group ERP for OCD. A combined treatment that also targets early maladaptive schemas may be a more effective approach for OCD patients with elevated EMS who don’t respond to standard ERP.

## Background

Obsessive-compulsive disorder (OCD) is a common disorder [1] that typically involves distressing, repetitive thoughts that are often accompanied by compulsive behaviors [2].

Cognitive behavioral therapy (CBT) that includes exposure and response prevention (ERP) is the psychological treatment of choice for OCD [3]. Although 60–70% of patients improve with CBT [4], only approximately 25% of patients meet criteria for full recovery post-treatment [5], and relapse rates range broadly from 0 to 50% [6]. Given that many patients do not recover with CBT, it is crucial to investigate the characteristics of patients who respond compared to those who do not. Increased knowledge about predictors of CBT response could reveal opportunities to modify treatment to improve outcomes.

Previous research identifying predictors of treatment outcome for OCD is mainly derived from patients given individual CBT. Additionally, most studies examining predictors of CBT outcomes are based on pre- to post-treatment change or predictors of change after a short-term follow-up period [7, 8]. Overall, reviews of the short-term outcome literature on CBT for OCD reveal inconsistent data on a range of predictors, but high levels of pre-treatment OCD severity and co-occurring depression are generally found to negatively influence short-term CBT outcomes [7, 8]. Predictor studies of longer-term outcomes (e.g., 5 or more years post-treatment) of CBT, pharmacotherapy or a combination for OCD are rare, and significant predictors vary from study to study [9–15].

Of particular relevance to the current report, co-occurring personality disorders (PD) have been identified as negative predictors of CBT outcomes for OCD. In shorter-term studies of CBT for OCD, comorbidity with Cluster A, schizotypal PD, narcissistic PD and having two or more PD’s have been associated with poorer outcomes [16]. One longer-term study found that comorbidity with obsessive-compulsive PD was also associated with poorer CBT outcomes [9]. Finally, pre-treatment schizotypal features [17] and higher neuroticism scores [11] have been linked to lower remission rates 5 to 6 years after initial treatment. These findings notwithstanding, it is important to note that not all studies have found that PDs have a negative impact on CBT outcomes [18].

Given the likely negative impact of at least some types of personality pathology on OCD treatment outcomes and inconsistencies in the literature related to the influence of personality pathology on CBT for OCD, it is important to further investigate the impact of personality-related variables on CBT response. One promising area of investigation relates to the impact of early maladaptive schemas (EMSs) on OCD symptoms and treatment outcomes. Young [19, 20] has proposed that personality pathology and recurrent symptom disorders can be in part explained by the concept of EMS. EMSs are defined as “a broad, pervasive theme or pattern comprised of memories, emotions, cognitions, and bodily sensations regarding oneself and one’s relationships with others developed during childhood or adolescence elaborated throughout one’s lifetime and dysfunctional to a significant degree” ([19], p., 7). EMSs are thought to develop through a combination of biological factors and unmet needs and experiences in the early years up to adolescence. Young has identified 15 different EMSs that are clustered in 5 categories called *schema domains* [21] (see Table 1).

**Table 1:** The 15 EMSs and the 5 schema domains of the YSQ-SF

Schema domains and early maladaptive schemas (EMSs)	Description
*Disconnection and Rejection*	*Trouble obtaining stable and safe attachment to significant others. Persons with high scores in this domain may have experienced a traumatic childhood that, in adulthood, causes repeated unstable relationships or avoidance of close relationships.*
Emotional Deprivation	The belief that others will not give emotional support
Abandonment/Instability	The belief that important others will leave
Mistrust/Abuse	The belief that one will be exploited by others
Social Isolation/Alienation	The assumption of not belonging to others
Defectivness/Shame	The belief of being worthless to others
*Impaired Autonomy and Performance*	*Difficulty functioning independently of others at same age. Persons with high scores in this domain may have experienced over-involvement from their parents in childhood and, in adulthood, may have difficulty mastering requirements and goals.*
Failure	The belief that one is incompetent compared to others
Dependence/Incompetence	The assumption that one can’t take care of oneself
Vulnerability to harm and illness	Expectation that an accident or illness is imminent
Enmeshment/Undeveloped Self	The feeling of fusion identity with important others
*Other-Directedness*	*Tend to emphasize other’s needs and feelings at the expense of their own. Persons with high scores in this domain may not have experienced unconditional acceptance in childhood and in adulthood, they may be more likely to set aside their needs in favor of others’ needs.*
Subjugation	The feeling that other’s needs are more important
Self-Sacrifice	Attention to other needs at the expense of oneself
*Overvigilance and Inhibition*	*Strict control over own feelings and unrealistic high demands on oneself. In childhood, persons with a high score on this domain may have learned to pay more attention to danger compared to pursuing happiness, thus increasing levels of pessimism and worry in adulthood*.
Emotional Inhibition	The assumption that one must not show emotions
Unrelented Standards/Hypercriticalness	The belief that one should do everything perfect
*Impaired Limits*	*Difficulty in respecting the feelings and needs of others. Persons with high scores in this domain may have experienced limited rules and responsibilities in childhood, and as adults, may have difficulty with impulse control.*
Entitlement/Grandiosity	The belief of being superior to others
Insufficient self-control/Self-Discipline	Lack of self-control and low frustration tolerance

The Table 1 is derived from Young [19]

The identification of EMS has contributed to the development of improved treatment models for patients with severe PDs (e.g., borderline PD) [19, 22]. Schema therapy (ST) [19, 20] was developed for patients with personality disorders and chronic psychiatric conditions who did not respond to traditional CBT. ST is an integrative therapy combining a variety of treatment models and theoretical approaches like cognitive behavioral therapy, psychodynamic therapy, attachment theory and developmental psychology. The main goal of ST is to modify the EMSs and coping strategies that maintain the schemas.

More recently, the impact of EMSs on a range of mental disorders, including OCD, has been investigated [23, 24]. Descriptive studies show that OCD patients have elevated EMSs compared both to healthy controls [25–27] and to patients with a range of other mental disorders [28, 29]. Only three studies have explored the impact of EMSs on pre- to post-treatment outcome for patients with OCD [30–32]. In the first study, Haaland et al. [30] identified *Abandonment/Instability* as a negative pre-treatment predictor of outcome, using a combined sample of patients given either group or individual CBT. Conversely, they found that high pre-treatment levels of *Self-sacrifice* predicted a positive outcome. Finally, they found that reductions in the *Failure* EMS during treatment predicted better post-treatment outcomes. In a second study on EMS and OCD treatment outcomes, Thiel et al. [31] reported that *Failure* and *Emotional inhibition* were negative predictors of CBT response. Finally, a third study involving OCD patients receiving individual cognitive therapy without ERP, found that the *Dependency/incompetence* EMS significantly mediated reductions in OCD symptoms over time [32]. Although these previous reports are valuable, there is still inconsistency regarding specific pre-treatment EMSs or schema domains that are significant associated with OCD treatment outcomes. The reasons for these inconsistencies might be explained by small sample sizes, differences in patient samples (e.g., inpatients, outpatients), variations in CBT treatments (e.g., CBT with or without ERP, individual CBT, group CBT) and/or overall length of treatment. Still, the EMS *Failure* seems to function as both a pre-treatment predictor and moderator of change over time, explaining between 18 and 21% of change with CBT for OCD [30, 31].

Personality-related factors, including EMSs, are generally thought to be relatively stable throughout the life-course [19, 33] and thus may be particularly likely to have an impact on OCD outcomes over time. However, no study has to our knowledge examined the impact of EMSs on OCD treatment-outcome beyond the post-treatment assessment point. So far, we know little about whether OCD patients who relapse or do not recover in the *long-term* have higher EMSs at pre-treatment. Understanding the potential impact of pre-treatment EMSs on long-term CBT outcomes could inform treatment modifications for OCD patients with elevated pre-treatment EMSs.

Building on this background, in the current study, we examined the impact of baseline EMSs on group ERP for OCD patients who were followed up several years after completing treatment. The present study addresses three research questions: 1) Do overall pre-treatment ratings of EMS predict OCD symptoms many years later? 2) Is there a difference in pre-treatment overall EMS ratings between patients who achieved status as *recovered* versus those who did *not recover* at different assessment points? and 3) Is there any difference in overall EMS changes over time between patients who recovered versus patients who still had clinically significant OCD symptoms at long-term follow-up? Beyond these specific research questions, we also investigated the impact of pre-treatment ratings of specific EMSs on change in OCD symptom severity in the long-term as an exploratory aim.

Our central hypothesis in this paper is that patients with no response to treatment or patients who relapsed during the follow-up period would be more likely to have elevated pre-treatment EMSs compared to those who responded to initial treatment and maintained gains over time.

## Method

### Participants and procedure

A total of 65 patients were invited to participate in this observational long-term follow-up study. All potential participants were treatment-seeking before they completed a manualized group ERP program for OCD [34] in a general outpatient clinic 5 to 11 years prior to participating in this study. The therapy was conducted in groups of six patients with two therapists. The groups met weekly for 12 weeks with each session lasting 2.5 h. All participants had a primary diagnosis of OCD before starting treatment. This original sample has been described in two previously published reports [35, 36].

Forty (*n* = 40) of the 65 eligible patients agreed to participate in the current long-term follow-up study. The remaining 25 patients either refused to participate (*n* = 24) or were not traceable (*n* = 1). The Regional Committee for Medical and Health Research Ethics^1^ approved this study. All participants gave written informed consent before taking part in the current long-term outcome study. All participants in the current study were previously assessed at pre-treatment, post-treatment and 12-months following the original group ERP program. For the present study, participants were assessed at mean of 8.23 years (*SD* = 1.86) after completing treatment (from now on referred to as “extended follow-up”). Twenty-four (61.5%) of these participants reported that they had received additional OCD treatment (i.e., additional ERP; therapy not involving ERP; anti-obsessional medication) between completion of group ERP and the extended follow-up assessment. The mean age of the participants at the extended follow-up was 43.6 years and 77.5% were female. The mean Y-BOCS total score at pre-treatment was 23.15 (*SD* = 3.63), indicating moderate- to severe OCD symptoms. More information about participant characteristics (marital- and employment-status) and additional study procedures are described elsewhere [36].

### Measures

**The Yale-Brown Obsessive Compulsive Scale** (Y-BOCS: [37, 38]). Obsessive-compulsive symptoms were assessed with the Y-BOCS. The Y-BOCS is a 10-item interview that provides sub-scores for obsessions, compulsions and a total score of OCD severity. The total score ranges from 0 to 40, with higher scores indicating greater OCD severity. The Y-BOCS has been documented as a reliable and valid tool [39].

**Young Schema Questionnaire – Short Form** (YSQ-SF: [21]). The Young Schema Questionnaire (YSQ) is a self-report inventory developed to assess underlying EMSs [40]. In the current study we used the original short version that contains 75 items and assess 15 specific early maladaptive schemas developed from the original long version of YSQ, which consist of 205 items [40]. Similar psychometric properties, validity and levels of clinical utility are reported between YSQ-SF and the long version of YSQ [41]. EMSs are organized in five s*chema domains* in YSQ. The five schema domains are; (1) *Disconnection and Rejection* (trouble getting stable and safe attachment to significant others), (2) *Impaired Limits* (difficulty in respecting the feelings and needs of others), (3) *Other-Directedness* (tendency to emphasize other’s needs and feelings on the expense of their own), (4) *Overvigilance and Inhibition* (strict control over one’s own feelings and unrealistic high demands on oneself) and (5) *Impaired Autonomy and Performance* (difficulty functioning independently of others at same age). Each item is rated using a 6-point Likert scale from 1 = “completely untrue of me” to 6 = “describes me perfectly”. There are five questions for each of the specific 15 EMSs (see Table 1). The total sum (YSQ-SF total score) is the addition of all raw item scores divided by 75. YSQ-SF is widely used across cultures and translations (e.g. Canada: [42], Belgium [43];, Spain [44], Britain [41], Australia and South-Korea [45]) and has shown good to excellent consistency both for the YSQ total score and the individual EMSs in Norwegian samples [46, 47]. The Norwegian version of YSQ-SF has been translated back to English with no substantial differences in meaning from the American version for the 75-item scale [46]. YSQ-scores have shown discriminant validity between clinical populations in Norway [33, 46, 48] and between clinical and non-clinical samples [33]. Test-retest reliability of YSQ-SF in a Norwegian study has been shown to be satisfactory, with a mean duration of 72 days [46]. Finally, in the current study, Cronbach’s alpha for YSQ-SF total scores ranged from 0.97 to 0.99 at the four measurement occasions, indicating excellent internal consistency [49].

**Beck Depression Inventory** (BDI: [50]) is a self-report inventory for depression symptoms, consisting of 21 items rated on a four-point Likert scale, ranging from 0 (“not at all”) to 3 (“severe”). The BDI has good psychometric properties [51]. In the current sample, Cronbach’s alpha for BDI were excellent, ranging from 0.92 to 0.94.

### Definition of recovery

To define the status as recovered, we used the same classification [52] as in the original study by Haaland et al. [35]. To be classified as recovered, participants must have a post-treatment Y-BOCS total score of 14 or less and must have improved at least 8 points from pre to post-treatment. Sixteen (40%) patients achieved recovered status at the extended follow-up whereas 24 patients (60%) did not [36].

### Sample size determination

We assumed that 50% of the invited participants (*N* = 65) would be classified as recovered at the extended follow-up based on data from the previously published 12-month follow-up study by Haaland et al. [35]. Moreover, we expected that most patients would be willing to participate in the extended follow-up, which would likely yield a sample of 25 participants in both the recovered and the non-recovered groups. Statistical power analysis performed with regard to the detection of differences between the groups on the potential predictor variables showed that with an α-level at 0.05 and a β-level at 0.20, 50 participants would be sufficient to detect differences of a moderate effect size (Cohens *d* = 0.80) with a two tailed t-test.

With respect to YSQ-SF total score, the legitimacy of these analyses was supported by data from Thiel et al. [31] who found a difference of .3 between responders and non-responders and a pooled SD of .75 at post-treatment. At the extended follow-up in our study, it is to be expected that the difference between recovered and non-recovered patients would be considerably larger, but we used a conservative estimate of .60 in the analyses. Using these numbers in a power calculation gave an effect size of .80 and a power of .80 as well. With a reduction from 50 to 40 participants, power diminished from .80 to .70, which we considered to be adequate to perform the predictor analyses since the difference of .60 is a very conservative estimate.

### Data analyses

The patterns of missing values for YSQ-SF protocols were examined. Independent t-tests were used to compare pre-treatment measures between patients who were recovered versus those who did not recover at the various measurement points. In addition, independent t-tests were used to compare the pre-treatment YSQ-SF total score of those who relapsed or had a delayed remission with those who were either unchanged or met criteria as recovered across all four measurement points (pre- and post-treatment, 12-month and the extended-follow-up). Fisher’s exact tests and independent sample t-tests were used to investigate differences in demographic variables between participants who were recovered and participants who were not recovered at the extended follow-up. All tests of differences were performed as two-tailed, unless otherwise noted.

Multiple regression analysis was conducted for predicting outcome with the Y-BOCS at extended follow-up as the independent variable. In the regression analysis we explored whether pre-treatment YSQ-SF total score was related to the extended follow-up outcome measured by Y-BOCS. Residual plots and histograms for residuals were checked which showed normal distribution of the residuals.

The third research question was addressed by the mixed models procedures in SPSS. YSQ-SF total score was entered as the outcome (dependent) variable. “Time” and “group” were entered as covariates, as well as the interaction between “time” and “group”. Time was coded as 0, 1, 2, and 3, corresponding to the four measurement occasions (pre-treatment, post-treatment, 12-month follow-up and 8-year follow-up). “Group” is a dummy variable indicating patients who were recovered versus patients who were not recovered from their OCD. Log likelihood estimation (LLH) and Akaike Information Criterion (AIC) were used to evaluate model fit.

The first step in the modelling procedures was to model the mean structure of the data [53], in which only “time” was included (LLH = 342, AIC = 354). The second step was to include a random intercept, which implies that each patient was assigned an individual estimate of the YSQ-SF total score at pre-treatment. This step also included the modelling of the covariance structure of residuals, comparing a diagonal covariance matrix with an autoregressive covariance matrix. The second step resulted in a considerably better model fit (LLH = 201, AIC = 211). In the third step, we compared a pure linear model (a straight regression line through all measurement occasions) with a linear spline model with the knot at post-treatment. This model implies that change trajectories were analyzed for two different time periods within the same model, i.e., from pre-treatment to post-treatment (where the knot was positioned) and from post-treatment to extended follow-up (through 12-month follow-up). The model fit did not change as compared with the second model (LLH = 200, AIC = 212). The fourth and last step was to include the interaction with “time” and “group” in the simple linear model as well as in the linear spline model to examine whether recovered patients had different change rates than non-recovered patients with respect to EMS. The inclusion of the “time by group” interaction in the simple linear model yielded a slight improvement of model fit (LLH = 192, AIC = 206). However, the linear spline model did not improve by including this interaction (LLH = 191, AIC = 209).

Effect sizes for the YSQ-SF total scores were calculated utilizing a formula derived from Cohen [54].^2^ To reduce the risk of Type I errors due to multiple comparisons, a Benjamini-Hochberg procedure with false discovery rate was set at 10% for all analysis [55]. These methods are in line with the recommendation of the American Statistical Association (ASA) [56]. The Benjamini-Hocberg procedure was preferred above the Bonferroni correction because of risk for type II errors with the small sample sizes [57] in the present study. *P*-value in the current study was 0.045 corresponding to a *p*-value of 0.05 for a single comparison. All statistics were calculated using IBM SPSS version 23.0 [58] except Benjamini-Hocberg procedure [55] that was performed by hand.

### Collinearity statistics

Multicollinearity among predictor variables was statistically investigated by computing variance inflation factors (VIF). The highest VIF was 1.67 which is far below the suggested cut-off value of 10 that indicates a collinearity problem [59]. As a further part of the multicollinearity examination, correlation coefficients were conducted among predictor variables. A linear regression analyses assume that the degree of correlation should not exceed 0.90 due to problems with multicollinearity [60]. As shown in Table 2, a significant moderate degree of correlation was observed between the dependent variable and the pre-treatment YSQ-SF total score. A moderate to high degree of correlation was found between the YSQ-SF total score and the two other independent variables, BDI and Y-BOCS, indicating that problems with multicollinearity are not a serious problem in the current study.

**Table 2:** Correlations between pre-treatment Y-BOCS, BDI and YSQ-SF total score and Y-BOCS at the extended follow-up

	1	2	3	4
1 Y-BOCS extended FU^a^				
2 Y-BOCS pre-test	.30			
3 BDI pre-test	.16	.27		
5 YSQ-total pre-test	.36*	.50**	.51**	

*FU*^a^ Follow-up. *Correlation is significant at *p* < 0.05. **Correlation is significant at *p* < 0.01

### Missing data

Sixteen YSQ-SF protocols were totally missing and 3 more protocols were excluded due to missing items. Taken together; 3, 5, 6 and 5 of the YSQ-protocols were missing at respectively pre-test, post-test, 12-month and the extended follow-up. Little’s MCAR test was run on the remaining data, and it indicated that data was not missing *completely* at random (MCAR). However, when the pattern of missing variables was closely examined, using the method described by Little and Rubin [61], it was not possible to find any definitive pattern. Therefore, we proceeded with analysis treating the data as missing at random (MAR). Of the remaining YSQ-SF protocols, 0.3% of the items were missing for the four measurement points. One participant had 19 of 75 items that were missing due to data management issues. This participant was excluded, and missing data for YSQ-SF was by this reduced to 0.2%. To reduce missing data for the YSQ-SF total score, mean imputation was applied to individual scale items when fewer than 5% of items were unanswered [62]. Single EMSs were excluded if any items were missing.

## Results

### Comparison of additional treatment between the recovered versus the not recovered group in the follow-up period

Ten participants (62.5%) in the recovered group and 14 participants (58.33%) in the non-recovered group reported that they had received additional treatment between the initial group ERP and the extended follow-up. There were no significant differences between the recovered group and non-recovered group regarding whether they received additional treatment of any kind or whether they received a specific type of treatment. The largest difference between the groups was that nine of the 24 (37.5%) participants in the non-recovered group and two (12.5%) of the participants in the recovered group received additional ERP for OCD in the time period between the end of the original group CBT and the long term follow-up assessment point [*M* = 0.41 (0.50) vs. *M* = 0.14 (.36); *t* (34) = 1.84, *p* = .075].

### Comparison of demographics between the recovered versus the not recovered group at the extended follow-up

There were no significant differences with respect to gender (*p* = 1.00), age [*M* = 43.56 (11.62) vs. *M* = 40.91 (13.52); *t* (37) = .637, *p* = .528], marital status (married and cohabiting versus single, divorced and separated) (*p =* .728) and employment status (employed, student, retired versus unemployed, homemaker and disabled) (*p* = .514) between the recovered and the non-recovered group.

### YSQ-SF total score for follow-up participants versus non-participants

To control for selection bias, the YSQ-SF total scores were compared between the group (*n* = 40) that agreed to participate in this long-term follow-up study and those who were contacted but refused to participate (*n* = 25). There were no significant differences at pre-treatment [*M* = 2.36 (0.84) vs. *M* = 2.41 (0.67); *t*(58) = 0.228, *p* = .821], post-treatment [*M* = 2.18 (0.82) vs. *M* = 2.10 (0.72); *t*(52) = 0.075, *p* = .941] and change scores at post-treatment [*M* = 0.19 (0.45) vs. *M* = 0.21 (0.49); *t*(49) = 0.122, *p* = .903] between these groups.

### Research question 1: predictive value of the pre-treatment YSQ-SF total score and OCD symptom severity at the extended follow-up

We investigated the factors that most influenced the OCD symptom severity at the extended follow-up. The dependent variable was the total Y-BOCS score at the extended follow-up time point. Due to the limited sample size (*N* = 40), only 3 independent factors measured at pre-treatment were included in the multiple regression analysis: 1) OCD symptom severity (Y-BOCS), 2) Depression symptoms (BDI), and 3) Maladaptive schema total score (YSQ-SF). In the first step, the three independent variables were entered one by one in a single linear regression analysis. Only the YSQ-SF total score was significantly related to the dependent variable (*p* = 0.029, adjusted R square = .105). To investigate whether the three independent variables adjusted for each other, thus giving better predictive ability, they were entered together in a backward elimination multiple-regression analysis (see Table 3). This analysis showed no change from the simple regression analysis. YSQ-SF total score at pre-treatment explained 10.5% of the obsessive-compulsive symptoms at the extended follow-up.

**Table 3:** Multiple backward elimination regression analysis using Y-BOCS at the extended follow-up as dependent variable

Factors	Beta	*T*	*P*	*R* ^2^
Model 1				0.052
Y-BOCS pre	0.046	0.246	0.807	
BDI pre	0.022	0.115	0.909	
YSQ-SF total pre	0.326	1.552	0.130	
Model 2				0.080
Y-BOCS pre	0.047	0.253	0.802	
YSQ-SF total pre	0.336	1.818	0.078	
Model 3				0.105
YSQ-SF total pre	0.360	2.828	0.029*	

*Correlation is significant at *p* < 0.05, *Y-BOCS pre* Yale-Brown Obsessive-Compulsive Scale, pre-treatment scores, *BDI pre* Beck Depression Inventory, pre-treatment scores, *YSQ-SF total pre* The Young Schema Questionnaire - Short Form, total pre-treatment scores

According to the best estimate of the regression line for Y-BOCS at the extended follow-up, an unstandardized B of 3.065 indicates that on average, an increase of 1 unit in YSQ-SF total score is associated with approximately a 3 unit increase in Y-BOCS at the extended follow-up.

### Research question 2: differences in pre-treatment YSQ-SF total score between those who recovered and those who not recovered at post-test, 12-month and the extended-follow-up, respectively

Independent sample t-tests showed that the non-recovered group at the extended follow-up had significant higher pre-treatment YSQ-SF total score, as well as BDI scores, compared to the recovery group (Table 4). These differences were not significant between patients who were recovered versus not recovered at post-test and 12-month follow-up, respectively. There were no significant differences in pre-treatment OCD severity between the recovered and not-recovered groups neither at post-treatment, 12-month nor the extended follow-up time points.

**Table 4:** Comparing pre-treatment scores between the recovered and non-recovered patients at post-treatment, 12-month- and the extended follow-up

Recovery status at three measure-points	Measures at pre-test	N	Mean (SD) Recovered	N	Mean (SD) Non-recovered	t	Sig. 2 tailed
Post-treatment	YSQ-SF total score	13	2.09 (0.79)	24^a^	2.53(0.86)	1.52	ns.
	BDI	13	15.15 (8.06)	27	16.95 (12.08)	0.56	ns.
	Y-BOCS	13	23.69 (2.90)	27	22.89 (3.96)	−0.73	ns.
12-month F.U.^d^	YSQ-SF total score	13^a^	2.14 (0.68)	18	2.59 (0.85)	1.59	ns.
	BDI	16	16.17 (9.80)	18	17.31 (13.05)	0.29	ns.
	Y-BOCS	16	22.78 (2.29)	18	22.63 (4.32)	−0.13	ns.
Extended F.U.^d^	YSQ-SF total score	15^b^	1.91 (0.52)	22^c^	2.69 (0.90)	3.33	0.02*
	BDI	16	12.31 (8.53)	24	19.08 (11.53)	2.13	0.04*
	Y-BOCS	16	22.50 (2.78)	24	23.58 (4.10)	0.97	ns.

^a^ = 3 YSQ-SF protocols missing, ^b^ = 1 YSQ-SF protocols missing and ^c^ = 2 YSQ-SF protocols missing, ^d^ = Follow-up, * = Correlation is significant at *p* < 0.05 and *ns* not significant

A closer analysis showed that 19 (47.5%) of the participants had changed their status between recovered and non-recovered from post-test to the extended follow-up. Eleven participants (27.5%) had relapsed between post-test and the extended follow-up and had significantly higher YSQ-SF total score at pre-treatment compared to those who were recovered at the extended follow-up [*M* = 2.77 (0.85) vs. *M* = 1.91 (0.52); *t*(24) = 2.98, *p* = .009, Cohen’s *d* = 1.19]. In addition, eight participants who had remission later than post-test had a significant lower YSQ-SF total score at pre-treatment compared to those that were non- recovered [*M* = 2.05 (0.57) vs. *M* = 2.69 (0.89); *t*(27) = 2.21, *p* = .042, Cohen’s *d* = 0.86].

### Research question 3: change in YSQ-SF total scores from pre-treatment to through all measurement points

**Fig. 1:**
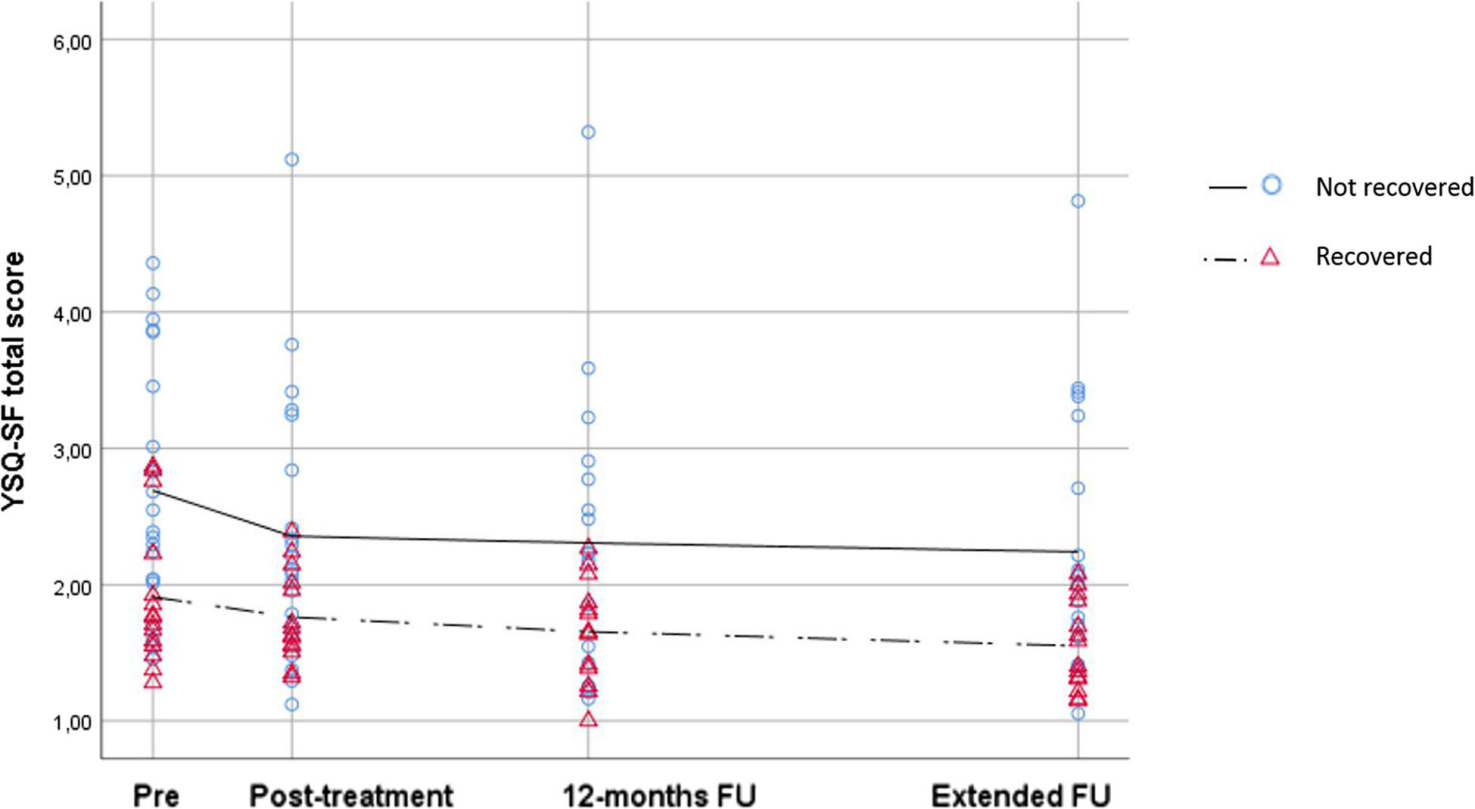
Clinical course of YSQ-SF total score for recovered versus non-recovered patients at the extended follow-up

Figure 1 displays the YSQ-SF total score change profiles for recovered and non-recovered patients. It can be seen that the decrease in YSQ-SF total scores was most pronounced from pre-treatment to post-treatment for both groups. Moreover, the non-recovered group had higher YSQ-SF total scores at pre-treatment. This difference remained stable over time.

Indeed, the results of linear mixed models analyses indicated that there was a slight but significant decrease in YSQ-SF total score from pre-treatment to extended follow-up for the entire sample (decrease of .14 points per measurement occasion, *t* = 4.6, *p* = .000). Furthermore, the change in YSQ-SF total score was greater from pre- to post-treatment than from post-treatment to the extended follow-up period for the entire sample. A linear spline model with a knot at post-treatment showed that the decrease from pre-treatment to post-treatment was larger (.22 points, *t* = 3.0, *p* = .003) than the decrease from post-treatment through 12-months follow-up to extended follow-up (.11 points per interval, *t* = 2.5, *p* = .017).

The linear model including the “time” by “group” interaction showed that the non-recovered group had higher YSQ-SF total scores at pre-treatment than the recovered group (*t* = 2.9, *p* = .005). However, EMS scores decreased at the same rate in both groups (*t* = .635, *p* = .529). The most complex model, the one in which the “time” by “group” interaction was included for two different time periods, showed similar results finding no difference in YSQ-SF total score change rates across the two groups, not from pre-treatment to post-treatment, neither from post-treatment to extended follow-up.

#### Outlier analyses

Inspection of the residuals plot showed that one patient in the non-recovered group had very high YSQ-SF total scores at the follow-up investigations (see Fig. 1). Mixed models analyses without this patient did not change the overall results. In the simple linear model, the change rate became somewhat larger (.16 points per occasion, *t* = 5.7, *p* = .000), and in the interaction model, the time x group interaction remained insignificant (*t* = 1.2, *p* = .219).

### Effect sizes for change in YSQ-SF total score

We followed Cohen’s [54] proposal to classify effect sizes as small (0.20–0.49), medium (0.50–0.79) or large (0.80 and above) in improvement in the YSQ-total score. For the entire sample the effect size (Cohens *d*) was small from pre- to post-test (*d* = 0.30), and remained small through the 12-month follow-up (*d* = 0.41) and to the extended follow-up (*d* = 0.48). Both the non-recovered (*d* = 0.37) and recovered group (*d* = 0.34) also had a small effect size for pre- to post-test. However, for the recovered group the effect size was medium (*d* = 0.57) at 12-month follow-up and large (*d* = 0.83) at the extended follow-up. For the non-recovered group, the effect size was still small (*d* = 0.39) at 12-month follow-up and medium (*d* = 0.50) at the extended follow-up.

### The impact of specific pre-treatment EMSs on long-term OCD symptom severity as an exploratory aim

To obtain more detailed information of the relationship between *specific* pre-treatment EMSs and extended follow-up outcomes, independent sample t-tests were conducted for the 15 EMSs. Table 5 shows that 10 of the 15 schemas (Abandonment/Instability, Emotional Deprivation, Mistrust/Abuse, Social Isolation/Alienation, Defectiveness/Shame, Dependence/Incompetence, Vulnerability to Harm and Illness, Subjugation, Emotional inhibition and Entitlement/Grandiosity) at pre-treatment were significant lower in the recovered group at the extended follow-up comparing to those who were not recovered. Five of these 10 schemas belong to the domain *Disconnection and Rejection* while the remaining five EMSs were evenly distributed on the other four domains. In addition, there were also a trend toward lower values on the remaining five EMSs, but these were not significantly different between the groups.

**Table 5:** Comparing the 15 pre-treatment EMSs between recovered and non-recovered patients at the extended follow-up

Domains and EMS	Mean (SD) Recovered	Mean (SD) Not-recovered	*t*	Sig. 2 tailed
Disconnection and Rejection				
Abandonment/Instability	1.76 (0.81)	2.62 (1.27)	2.53	.016*
Emotional Deprivation	1.29 (0.41)	2.20 (1.10)	3.51	.002**
Mistrust/Abuse	1.27 (0.28)	1.73 (0.84)	2.41	.023*
Social Isolation/Alienation	1.37 (0.48)	2.08 (1.28)	2.26	.025*
Defectiveness/Shame	1.32 (0.44)	2.15 (1.36)	2.67	.013*
Impaired Autonomy and Performance				
Failure	1.45 (0.51)	1.97 (1.16)	1.88	.070
Dependence/Incompetence	1.45 (0.51)	1.87 (0.88)	2.15	.039*
Vulnerability to Harm and Illness	1.32 (0.50)	2.05 (1.03)	2.86	.007*
Enmeshment/Undeveloped Self	1.51 (0.68)	2.05 (1.03)	1.80	.080
Other-Directedness				
Subjugation	1.38 (0.61)	2.08 (1.35)	2.13	.041*
Self-Sacrifice	2.21 (0.51)	2.82 (1.27)	1.99	.056
Overvigilance and Inhibition				
Emotional Inhibition	2.12 (0.51)	2.66 (0.88)	2.63	.024*
Unrelenting standards/Hypercriticalness	2.39 (1.08)	3.14 (1.30)	1.97	.058
Impaired Limits				
Entitlement/Grandiosity	1.50 (0.48)	2.03 (1.17)	2.69	.012*
Insufficient Self-Control/Self-Discipline	1.42 (0.49)	1.70 (0.63)	1.52	.138

*t* = independent t-test, *significant at *p* < 0.05, ** significant at *p* < 0.01

## Discussion

This study sought to expand the current knowledge of the relationship between early maladaptive schemas and OCD symptom severity using a longitudinal design. The key findings in this study are: 1) Pre-treatment YSQ-SF total score was significantly associated with more severe OCD symptoms at the extended follow-up compared to pre-treatment depression and obsessive compulsive symptom severity; 2) Non-recovered patients at extended follow-up had significantly higher scores on early maladaptive schemas (i.e. YSQ-SF total score) at pre-treatment compared to the recovered patients; and 3) Linear mixed model analysis showed that the largest reduction of YSQ-SF total score occurred from pre-treatment to post-treatment with smaller but still significant reductions from post-treatment to the extended follow-up both in the recovered- and the non-recovered groups.

As far as we know, this study is the first to show that pre-treatment early maladaptive schemas may be associated with OCD symptom severity many years after treatment. Earlier studies examining the role of early maladaptive schemas for OCD have had a pre- to post design and detected specific EMSs to be negative (e.g. Abandonment/Instability and Failure) or positive (Self-sacrifice) predictors of treatment outcome [30, 31].

Our analysis of individual EMS scores must be seen as exploratory given our limited sample size. This limitation notwithstanding, it is important to note that t-tests indicated that 10 of 15 pre-treatment YSQ-SF sub-scores were significantly higher in the non-recovered group compared to the recovered group. These findings suggest that a number of specific maladaptive schemas may negatively impact long-term outcomes after group ERP. Of particular interest is the fact that all five EMSs belonging to the *Disconnection and Rejection* domain (Abandonment/Instability/Instability, Emotional Deprivation, Mistrust/Abuse, Social Isolation/Alienation and Defectiveness/Shame) were significantly higher at pre-treatment in the non-recovered patients at the extended follow-up compared to recovery group. Support for this finding is also found in the study by Thiel et al. [31] who reported that three out of four baseline EMSs (Mistrust/Abuse, Social Isolation/Alienation and Defectiveness/Shame) were significantly higher in the group of patients categorized as non-responders after CBT with ERP for OCD. Indirect support is also found in a comparative study that found OCD patients, compared to normal controls, had significantly higher scores on two of three EMSs (Defectiveness/Shame and Social Isolation/Alienation) [26] after controlling for depression. According to Young [19], patients with elevated schemas in the domain *Disconnection and Rejection* have little ability to relate safely and satisfactorily to others and are often the most damaged (i.e., the most severe personal pathology). Schemas from this domain are also more prevalent among patients with borderline personality disorder [63]. Future long-term studies with larger samples are needed to definitively investigate the impact of specific EMSs on CBT for OCD.

Our finding that higher baseline EMSs was negatively associated with OCD symptom severity at extended follow-up is consistent with studies indicating that pre-treatment PDs are also related to poorer outcomes after CBT for OCD [9, 17]. Conversely, our findings that higher pre-treatment OCD and depression symptoms was not associated with OCD symptom severity at extended follow-up is in contrast to studies that have found them to be predictive of poorer CBT outcomes over the long-term [11, 12].

Although there are no other studies that have examined relationship between pre-treatment EMSs on very long-term treatment outcomes for OCD or for other anxiety disorders, there is one comparable long-term study for depression severity and episodes of Major Depression. In this study, two of five baseline schema domains, Undesirability (domain from the long version of YSQ) and Impaired Limits, predicted depression severity and number of major depressive episodes after 9 years, respectively [64]. Our results add to this limited literature suggesting that elevated pre-treatment EMSs may be an important negative indicator of the long-term course for a range of mental disorders.

It is interesting to note that although significant differences in pre-treatment YSQ-SF total score between participants who were recovered versus those not recovered were observed at extended follow-up, there were no significant differences in YSQ-SF scores between these groups at post-treatment or at 12-month follow-up. These results are indirectly supported by another long-term follow-up study finding that the presence of at least one comorbid PD did not predict outcomes for OCD patients in the short-term but were significant negative predictors 5 years after treatment [11]. A possible explanation for these results is that patients with high degree of personality pathology (i.e., high score on YSQ) may experience short-term reductions in their OCD symptoms after CBT but may struggle to maintain the improvement over time, due, at least in part, to their personality symptoms. This explanation is supported in the current study where we found that several of the patients who first benefited from treatment and later relapsed at the extended follow-up had higher pre-treatment EMSs. In addition, those patients who did not improve from pre- to post-treatment but were rated as recovered at any follow-up period, had significantly lower EMS scores at pre-treatment compared to those who never recovered. Rebound in OCD symptoms after successful ERP or comprehensive cognitive therapy among patients with certain concurrent personality traits or PDs has also been reported in other studies [65–67].

A related point of interest is our finding that YSQ-SF total score showed modest but significant decreases for the entire sample over time after a relatively brief group ERP. The most pronounced decrease in EMS for all participants was observed just after the active treatment but small significant reductions in YSQ-SF total score at 12-month and extended follow-up were also observed in both the recovered and non-recovered groups. It is important to note, however, that although significant, the effect sizes of these changes were small. Other studies have also found similar small, but significant changes in single EMSs from pre- to post- OCD treatment with ERP [30, 31] or cognitive therapy [32]. Modest decreases in EMSs have also been observed following brief CBT for social anxiety [68] and for other anxiety and depressive disorders [69]. Interestingly, the changes in YSQ-SF total score did not closely follow changes in OCD symptoms over time. Both the recovered and non-recovered groups had similar modest improvements in EMSs over time even though the recovered group experienced substantially greater decreases in OCD symptoms. In short, OCD improvement did not seem to have a proportional positive impact on maladaptive schema. Very small changes in EMSs over time for all participants and the lack of substantial changes in EMSs even for those who achieved large gains in OCD may not be surprising. According to Young [20] early maladaptive schema are deeply entrenched as the result of developmental processes and that they likely require intensive schema-focused therapy in order to make substantial changes in these beliefs.

### The limitations and strengths with this study

This study has limitations. For example, there was no control group, making it difficult to draw conclusions about treatment effects, especially since many participants had received additional treatment during the follow-up period [36]. The study was not designed to find causal relationships between reduction in EMSs and OCD symptoms. In addition, patients were treated with ERP which is not designed to change EMSs, but is specifically targeted toward improving OCD symptoms. Further, other potentially clinically important variables that have been shown previously to be significant predictors of outcome for OCD treatment have not been examined. For example, because of limited statistical power, hoarding pathology, increased anxiety, certain OCD symptom subtypes, unemployment, and being single/not married, were not included in the analysis, which is relevant because they have all been shown, at least in some studies, to be negative predictors for OCD treatment outcome [8]. As mentioned above, a shortcoming is also that the sample size did not allow for the systematic investigation of individual EMSs on outcomes over time. Future long-term outcome studies with larger samples would be very useful in addressing these important issues. Additionally, there is a substantial length of time between the 12-month follow-up and the extended follow-up periods. It would have been of interest to know more about the relationship between EMSs and OCD symptoms annually throughout the extended follow-up period. Annual assessments are particularly relevant for OCD given that waxing and waning of symptoms over time is commonly observed [70]. Finally, we did not assess for PDs in this study. Given that patients with personality psychopathology generally exhibit high EMSs [48], it is desirable to assess and control for PD comorbidity when studying relation between EMSs and OCD symptoms.

These weaknesses notwithstanding, a key strength with the current study is that we explore follow-up data for EMS at a mean of 8 years after OCD treatment. No other studies of CBT for OCD provide any follow-up EMS data beyond the post-treatment data point. Furthermore, contrary to some CBT studies for OCD [7, 71, 72], patients with severe PDs and/or major depression were included in this community-based project. Therefore, the participants in this study may be seen as more representative of typical treatment-seeking patients in routine outpatient clinics.

## Conclusions

This is the first study to longitudinally examine early maladaptive schemas’ impact on OCD symptom severity over the long-term. The results from the present study suggest that patients with higher pre-treatment EMS scores are less likely to recover in the long-term after receiving a 12-week course of group ERP for OCD. Poorer response to treatment among persons with high pre-treatment EMS scores is also underscored by the fact that many more of these patients sought additional treatment in the follow-up period compared to those with lower EMS scores.

A clinical implication may be that patients with high pre-treatment EMSs scores may benefit from a tailored treatment that targets both their OCD symptoms and personality-related problems. Special attention should be given to OCD patients with endorsed schemas in the domain *Disconnection and Rejection*. Particular focus on building a working alliance may be needed in this group of patients. Perhaps individual ERP may be a better approach than group ERP for these patients given that individual ERP allows for more time to develop a personal relationship with the therapist. It is also possible that persons with high *Disconnection and Rejection* EMSs may benefit from schema therapy or other clinical approaches that emphasize alliance building, either prior to or during exposure and response prevention. Promising results using a combination of schema therapy and CBT for non-responders, drop-outs or initial CBT refusers have been reported in two OCD case examples [73] and in an open trial of 10 OCD inpatients [74] suggesting that it might be advantageous for OCD patients with high pre-treatment EMSs as well.

## Data Availability

The datasets used and analyzed during the current study are available from the corresponding author on reasonable request.

## Abbreviations

AIC: Akaike Information Criterion
ASA: American Statistical Association
BDI: Beck Depression Inventory
CBT: Cognitive behavioral therapy
EMS: Early maladaptive schema
ERP: Exposure and response prevention
LLH: Log likelihood estimation
MAR: Missing at random
MCAR: Missing completely at random
OCD: Obsessive-compulsive disorder
PD: Personality disorder
ST: Schema Therapy
VIF: Variance inflation factors
Y-BOCS: Yale-Brown Obsessive Compulsive Scale
YSQ: Young Schema Questionnaire
YSQ-SF: Young Schema Questionnaire – Short Form

## Footnotes

1 REC number; 2013/ 1210 Sør-Øst

2 Cohen’s *d* = $$ \frac{M1-M2}{SD\mathrm{pooled}} $$
