# Correction to: Early maladaptive schemas impact on long-term outcome in patients treated with group behavioral therapy for obsessive-compulsive disorder

**DOI:** 10.1186/s12888-022-03776-8

**Published:** 2022-04-22

**Authors:** Tor Sunde, Benjamin Hummelen, Joseph A. Himle, Liv Tveit Walseth, Patrick A. Vogel, Gunvor Launes, Vegard Øksendal Haaland, Åshild Tellefsen Haaland

**Affiliations:** 1 DPS Solvang, Sørlandet Hospital, SSHF, Seviceboks 416, 4604 Kristiansand, Norway; 2 Clinic of Mental Health and Addiction, Oslo University Hospital, Oslo, Norway; 3 School of Social Work and School of Medicine-Psychiatry, University of Michigan, Ann Arbor, USA; 4 Department of Psychology, Norwegian University of Science and Technology, Trondheim, Norway; 5 Clinical Neuroscience Research Group, Department of Psychology, University of Oslo, Oslo, Norway

**Correction to: BMC Psychiatry 19, 318 (2019)**

**https://doi.org/10.1186/s12888-019-2285-2**

Following publication of the original article [1], the authors identified an error in Table 5. The correct table is given below.

**Table 5:** Comparing the 15 pre-treatment EMSs between recovered and non-recovered patients at the extended follow-up

Domains and EMS	Mean (SD) Recovered	Mean (SD) Not-recovered	*t*	Sig. 2 tailed
*Disconnection and Rejection*				
Abandonment/Instability	2.04 (0.97)	3.19 (1.49)	2.88	.007**
Emotional Deprivation	1.55 (0.75)	2.50 (1.23)	2.98	.005**
Mistrust/Abuse	1.51 (0.51)	2.22 (1.12)	2.64	.012*
Social Isolation/Alienation	1.87 (1.02)	2.79 (1.47)	2.26	.030*
Defectiveness/Shame	1.59 (1.29)	2.48 (1.42)	1.96	.058
*Impaired Autonomy and Performance*				
Failure	2.20 (1.46)	2.57 (1.31)	0.79	.435
Dependence/Incompetence	1.75 (0.92)	2.26 (1.04)	1.55	.130
Vulnerability to Harm and Illness	1.68 (0.85)	2.43 (1.09)	2.24	.032*
Enmeshment/Undeveloped Self	1.47 (0.63)	2.34 (1.36)	2.67	.012*
*Other-Directedness*				
Subjugation	1.73 (0.67)	2.96 (1.54)	3.36	.002**
Self-Sacrifice	2.87 (0.78)	3.52 (1.31)	1.90	.066
*Overvigilance and Inhibition*				
Emotional Inhibition	2.00 (0.99)	2.34 (1.34)	0.84	.405
Unrelenting standards/Hypercriticalness	2.99 (1.16)	3.64 (1.50)	1.42	.166
*Impaired Limits*				
Entitlement/Grandiosity	1.53 (0.55)	2.20 (1.00)	2.34	.025*
Insufficient Self-Control/Self-Discipline	1.92 (0.81)	2.50 (1.15)	1.70	.098

*Note*. *t* = independent t-test, *significant at *p* < 0.05, ** significant at *p* < 0.01

The original article [1] has been corrected.
